# Cost effectiveness of interpersonal community psychiatric treatment for people with long-term severe non-psychotic mental disorders: protocol of a multi-centre randomized controlled trial

**DOI:** 10.1186/s12888-015-0476-z

**Published:** 2015-05-02

**Authors:** Mark van Veen, Bauke Koekkoek, Niels Mulder, Debby Postulart, Eddy Adang, Steven Teerenstra, Lisette Schoonhoven, Theo van Achterberg

**Affiliations:** 1 Research Group Social Psychiatry & Mental Health Nursing, University of Applied Sciences Arnhem Nijmegen, PO Box 6960, Nijmegen, GL 6503 The Netherlands; 2 Altrecht Mental Health Services, Oude Arnhemseweg 260, Zeist, BK 3705 The Netherlands; 3 Pro Persona Mental Health Services, Wolfheze 2 ProCES, Renkum, BE 6874 The Netherlands; 4 Department of Psychiatry, Erasmus MC, University Medical Center, PO Box 2040, Rotterdam, CA 3000 The Netherlands; 5 Department of Research and Development, GGZ Oost Brabant Mental Health Services, PO Box 3, Boekel, ZG 5427 The Netherlands; 6 Epidemiology, Biostatistics & Health Technology Assessment, Radboud University Medical Center, Geert Grooteplein Zuid 10, Nijmegen, GA 6525 The Netherlands; 7 Scientific Institute for Quality of Healthcare, Radboud University Medical Center Nursing science, PO Box 9101, Nijmegen, HB 6500 The Netherlands; 8 Faculty of Health Sciences Highfield, Southampton, University of Southampton, SO17 1BJ Southampton, UK; 9 Centre for Health Services and Nursing Research, Kapucijnenvoer 35 blok d, box 7001, 3000 Leuven, Belgium

**Keywords:** Severe mental illness, Community mental health care, Long-term care, Cluster randomized controlled trial, Cost-effectiveness

## Abstract

**Background:**

This study aims for health gain and cost reduction in the care for people with long-term non-psychotic psychiatric disorders. Present care for this population has a limited evidence base, is often open ended, little effective, and expensive. Recent epidemiological data shows that 43.5% of the Dutch are affected by mental illness during their life. About 80% of all patients receiving mental health services (MHS) have one or more non-psychotic disorders. Particularly for this group, long-term treatment and care is poorly developed. Care As Usual (CAU) currently is a form of low-structured treatment/care. Interpersonal Community Psychiatric Treatment (ICPT) is a structured treatment for people with long-term, non-psychotic disorders, developed together with patients, professionals, and experts. ICPT uses a number of evidence-based techniques and was positively evaluated in a controlled pilot study.

**Methods/Design:**

Multi-centre cluster-randomized clinical trial: 36 professionals will be randomly allocated to either ICPT or CAU for an intervention period of 12 months, and a follow-up of 6 months. 180 Patients between 18–65 years of age will be included, who have been diagnosed with a non-psychotic psychiatric disorder (depressive, anxiety, personality or substance abuse disorder), have long-term (>2 years) or high care use (>1 outpatient contact per week or >2 crisis contacts per year or >1 inpatient admission per year), and who receive treatment in a specialized mental health care setting. The primary outcome variable is quality of life; secondary outcomes are costs, recovery, general mental health, therapeutic alliance, professional-perceived difficulty of patient, care needs and social contacts.

**Discussion:**

No RCT, nor cost-effectiveness study, has been conducted on ICPT so far. The empirical base for current CAU is weak, if not absent. This study will fill this void, and generate data needed to improve daily mental health care.

**Trial registration:**

Netherlands Trial Register (NTR): 3988. Registered 13th of May 2013.

## Background

In the Netherlands, as in many other developed countries, many people suffer from psychiatric disorders during their life. Recent epidemiological data show that 43.5% of the Dutch are affected by some form of mental illness during their life [1]. Depression (20.1%), anxiety (19.6%), and substance abuse (19.1%) have the highest lifetime prevalence: the first two appear in the Dutch top-4 of diseases with the highest disease burden [2]. Comorbidity with personality disorders, which have a prevalence of 9.1% in western society [3], results in poorer social functioning and limited recovery.

About 80% of all patients receiving mental health services (MHS) have one or more of the aforementioned non-psychotic disorders [4]. Between 16-18% of these patients do not respond well to short-term treatment (i.e. <15 contacts or <1 year treatment) and end up in long-term care [5,6]. Long-term treatment and care are poorly developed: 50-70% of these patients receive a form of long-term supportive treatment/counselling/care, which we refer to as care as usual (CAU). CAU currently is a low-structured treatment/care: biweekly contacts with a nurse, social worker or occupational therapist, in which daily issues are discussed [7]. The other 30-50% of the patients receive long-term psychotherapy – of which many are eventually referred to long-term CAU. Thus, often when short-term treatment has proven ineffective, long-term care with a poor focus is the only alternative. Specific treatments for subgroups, e.g. patients with chronic depression, exist [8] but are not widely implemented. As a result, large numbers of people yearly receive a non-descript form of long-term care. The lack of direction in CAU results in: 1) very long-term care (e.g. up to 10 years [9]) and 2) high care use. Several studies show that 10-30% of chronic patients use 50-80% of mental health care’s resources [10]. These resources include (intensive) ambulatory care, as well as services such as crisis intervention outside office hours, ambulance transport, and admissions to hospitals, and ER/ casualty-departments. Long-term and intensive care use is highly correlated with the perceived patient ‘difficulty’ [11,12]. When a patient is labeled ‘difficult’ quality of care often becomes low [13]. For patients this results in lower quality of life, more symptoms, and even higher care use [14].

For those patients who receive CAU, we developed Interpersonal Community Psychiatric Treatment (ICPT). Feasibility and preliminary effectiveness of ICPT were evaluated in a controlled pilot-study [9], in which ICPT was more successful than CAU on a number of outcome variables. Patients gained quality of life and social contacts, and used fewer health care services. Professionals (e.g. community psychiatric nurses and nurse specialists) valued the therapeutic alliance more positively, and experienced both patients and patient care as less ‘difficult’. ICPT is not yet standard care but is being used on a small scale. Given the positive outcomes in a group of patients with complex needs, ICPT seems a promising intervention. Yet data from RCTs on the (cost) effectiveness of ICPT is not available.

### Target population

The intervention in this study aims at a broad group of patients in terms of psychiatric diagnosis (non-psychotic disorders in several combinations) and in terms of demographic characteristics (although women, and persons with a lower socio-economic status are overrepresented), but a specific group in terms of care use (long-term and intensive). The severity of the disorder may account for the long duration of care, yet in psychiatric care people may also become accustomed to using services. Some studies highlight such iatrogenic dependency [15], and show very high service use of non-psychotic patients across health and social services [10]. We specifically aim at this group of patients, who have serious mental illnesses, but who may also have become accustomed to long term or high care use. These patients may be perceived as ‘difficult’ [16] and difficult-to-place, and be passed around by services [11]. They may get lost in the system, since they neither fit in long-term care programs (mostly aimed at patients with psychotic disorders), nor in short-term therapy (mostly aimed at patients with singular non-psychotic disorders, who respond well to medication and/or psychotherapy). Instead of keeping on ‘pampering and dithering’ we offer this group a generic program that aims at improving quality of life while decreasing costs.

### Research aim and hypotheses

This study aims at comparing the effectiveness and costs of ICPT in the treatment of people with long-term non-psychotic mental illness to CAU.

Based on a previous controlled pilot study of 36 patients [9], our main hypothesis is that ICPT is more effective in improving patients’ quality of life and social networks than CAU. Further, we hypothesize that ICPT is more effective in preventing or decreasing professionals’ perception of patients as ‘difficult’, resulting in higher quality of care than CAU and that ICPT is more effective in discharging patients to a lower level of care (i.e. general mental health care instead of specialized mental health care) and more cost-effective in reaching aforementioned clinical goals than CAU.

## Methods/Design

### Design

Multi-centre cluster-randomized clinical trial: participating professionals will be randomly allocated to either ICPT or CAU for an intervention period of 12 months, and a follow-up of 6 months (total 18 months). Participating patients will receive ICPT or CAU during 12 months. There is a measurement at baseline, an intermediate measurement (6 months after baseline), after the intervention period (12 months after baseline), and a follow-up measurement (6 months after end of intervention, 18 months after baseline).

### Randomization

The professionals (clusters) will be randomized the intervention (ICPT) or the control group (CAU) using randomized stratification by an independent statistician. The allocation sequences will be generated with an automated algorithm by a statistician independent from the recruiter of the professionals using a random sequence generation.

### Inclusion and exclusion criteria

Patients between 18–65 years of age with a presence of a non-psychotic psychiatric disorder such as depressive, anxiety and/or personality disorder and/or substance abuse and long-term treatment (>2 years) or high care use (>1 outpatient contact per week or >2 crisis contacts per year or >1 inpatient admission per year) in secondary mental health services will be included. Patients with a psychotic, bipolar I or cognitive disorder (e.g. dementia) and a lack of skill in understanding of, or communication in Dutch language are excluded.

**Figure 1:**
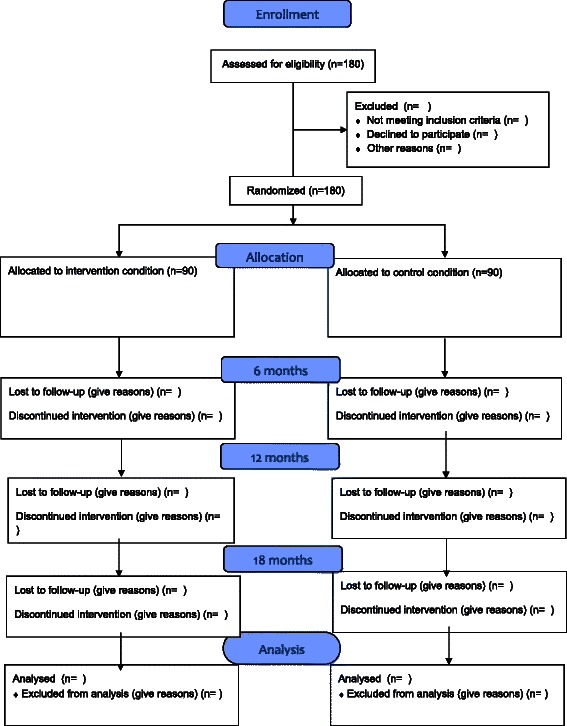
Participant Flow Chart.

Professionals who have an individual caseload of > 5 patients with a non-psychotic disorder, who are willing to be randomized to either CAU or the experimental ICPT-condition and have not expressed intention to leave the present service between now and 12 months are included. Refer to Figure 1 Participant flowchart for details.

### Sample size calculation

This study’s sample size calculation is based on the primary outcome variable, quality of life as measured with the Manchester Short Assessment of Quality of Life (MANSA), for which we found an effect size of 0.3 in our pilot study [17]. In a patient group in which quality of life is difficult to improve, an effect size of 0.3 signifies clinically meaningful progress. Furthermore we assumed a conservative Intra Cluster Correlation of 0.10 for clustering of patients, based on the scarce literature on the correlation between long-term psychiatric patients within one professional [17]. The correlation between baseline and follow-up measurement, also from our pilot study, was set at 0.5 and 0.8 for cluster and subject level, respectively. With an alpha of 0.05, and a power of 0.80, 36 clusters (professionals) of 5 participants (patients) each are required (total of 36 professionals and 180 patients needed for the analysis).

### Ethics

A certified Medical Ethics Review Committee, The Clinical Research Centre Nijmegen (CRCN), in The Netherlands has approved this study, registered under NL44744.091.13. This ethical approval covers all sites of data collection.

### Procedure

Three large mental health institutions participate in this study. Within these departments, professionals will be asked to participate in this study, and be randomized to either ICPT or CAU. Once a professional is included in the study, his or her patients meeting the inclusion criteria at patient level, will be informed about the study and be invited to participate. This invitation letter (to which a brochure about the research is attached), will be signed by the professional, and sent by the department’s management. Patients who express their willingness to participate may either contact their professional or the research team directly. The research team will then contact them by telephone or email, make an appointment for a face-to-face contact, and send formal information about participating in the study by post. In this face-to-face meeting the researcher will explain the study verbally, and obtain informed consent if the patient is indeed willing to participate.

### Treatment integrity

Treatment integrity in the experimental condition will be monitored and discussed by means of supervision. Since there is no clear treatment guideline for CAU, treatment integrity will not be monitored in the control group. Randomly selected audiotapes of treatment sessions will be evaluated by independent raters (Master-level students familiar with ICPT) masked to treatment condition. They will assess whether the tape is CAU or ICPT, and to which extent ICPT-elements are indeed used. The ICPT-professionals assess the ICPT-form and the attached scoring form after each session [9].

### Treatments

#### ICPT

Apart from various specific methods, the focus of ICPT very much lies on the participation of patients through attention for the interaction between patient, professional and social system. The match between patient and professional is highly important for the future course of the care process [17]. In ICPT, the patient is strongly encouraged to take responsibility for his or her recovery. Likewise, in the ICPT-training the professional is taught not to present him or herself as the all-knowing expert, but rather as a facilitator – yet within a clear frame and structure.

A number of stages were conceptualized in the intervention program, each fitting an important step in the theoretical model, resulting in three stages that fit the patient’s level of acceptance of help and cooperation. Apart from these stages (described in detail below), we concluded that an intervention for this patient group program should focus on: (1) a clear generic treatment structure (to prevent uninformed and haphazard low-dosage help), (2) a phased model (which fits the patient’s level of acceptance of help), (3) a therapeutic style that fits the phase the patient is in, (4) a routine monitoring of the interpersonal contact between patient and professional, and (5) support for team professionals [18].

I. **Generic structure:** Based on various evidence-based treatments of specific non-psychotic disorders [19,20], we introduced a fixed structure for each session, taking 45 minutes as the standard duration. The first 5 minutes are used by the professional and the patient to set a mutually agreed on agenda for the session. The next 5 minutes are used to look back from the current to the previous session. In the following 25–30 minutes the themes set on the agenda, are discussed and summarized. The last 5 minutes are used to look back on the session and to fill out a report form (professional) and a feedback form (patient).
II. **Stage model:** In the stage model patients may move from the 1st stage (optimization of working alliance), through the 2nd stage (clarification of an agreement on goals and tasks) to the 3rd stage (improvement of psychiatric and social functioning). In order to optimize the patient-professional interaction across all stages, it is crucial for the professional to determine in which stage the treatment contact is located. The stage model helps professionals to structure their treatment, using different methods across different stages.
III. **Therapeutic methods per stage:** One of the crucial elements of ICPT, in order to prevent ineffective illness behaviour and professional behaviour, is the differentiation of therapeutic styles across treatment stages. This approach is a variation of, but consistent with, the trans-theoretical model of change [21] which differentiates people’s readiness to change into various stages. Different methods (e.g. motivational interviewing) are used to prevent the usual mental health treatment ‘script’. In this script, the professional is the one who looks for problems in the patient and suggests improvements of his or her behaviour, while the patient is a passive recipient of help. In the second stage of ICPT, motivational interviewing is used to do enable systematic goal-setting. After an initial open question to focus the patient on the future, a widely used tool to assess care needs [22] is used, after which specific goals are jointly formulated. This careful process of mutual goal setting seeks to avoid common pitfalls: the patient feeling that treatment goals are forced upon him or her, and the professional feeling that urgent patient needs (e.g. financial problems) have not come under discussion. In the third stage of ICPT, three different goal-oriented methods are used to improve personal and social functioning. Practical case management, motivational interviewing and aspects of cognitive behaviour therapy may be used. This third stage of ICPT, which may not be reached by all patients, aims to offer practical help after goal-setting in stage two has been concluded.
IV. **Application of feedback forms:** In ICPT, both professional and patient fill out a form about the session they have just had. Both rate items on the Session Rating Scale [23], thereby informing one another on their (dis) content with the working alliance. In addition, professionals score in which stage of the treatment contact this session could be located, as well as which methods were used, if treatment goals were discussed, and a number of other elements, using the ICPT-form and scoring form after each session [9]. Patients, on the other hand, rate their own input in the session’s content. By these means, both parties are delegated responsibility for the working alliance and their substantive input in the session.
V. **Supervision:** Every two weeks, a team-wise supervision takes place in which a treatment situation of two different professionals is jointly analysed. We use a brief version of a supervision protocol that has been developed and evaluated in Dutch long-term mental health care [24].

#### CAU

Care as usual (CAU) currently is a low-structured treatment/care: biweekly contacts with a nurse, social worker or occupational therapist, in which daily issues are discussed [7]. This CAU lacks an empirical and theoretical base and may foster dependence and repeated crises through its ad-hoc character [25] and lack of clear aims [26]. Without a clear frame, this CAU turns into – politically incorrect – ‘pampering and dithering , reinforcing patients’ dependency and high care use [27]. The present CAU is offered by non-academically trained professionals (e.g. nurses and social workers) who have always relied on practical, day-to-day interventions in acute circumstances (e.g. locked units or psychosocial crises).

Although these generalizing statements do not apply to all of these professionals, most – if not all – of them feel that they lack a solid theoretical base from which to understand the disorder, its long (er) term character, and possible treatment.

Participating professionals in the experimental condition will receive a 4-day training in ICPT over 4 weeks’ time. The ICPT-training has been piloted twice before, and consists of the following elements: (1) theoretical overview (4 hours), (2) generic ICPT-skills, e.g. agenda setting (4 hours), (3) relationship management skills (8 hours), (3) motivational interviewing and goal setting skills (8 hours), (4) case-management skills and behavioural analysis skills (4 hours) and skills to discharge patients to a lower form of care. It combines lectures, group discussions, one-on-one and group-wise role-playing, homework assignments, and self-study of provided literature. Substantial effort is put in tailoring the training program to the needs and competencies of the participants. Some of the ICPT-methods for patients with non-psychotic disorders are aimed at Master-level professionals (e.g. Cognitive Behavioural Therapy), whereas the participating professionals – the key professionals of patients and also those intended to carry out ICPT –usually have Bachelor-level qualifications. Tailoring will be done by inviting specialists with extensive experience with both the target group of professionals, and the method to be taught. In following group-wise supervision sessions ICPT-skills will be practiced, and cases will be discussed.

### Measurements

#### Demographic variables

At baseline, participants complete questions concerning living situation, marital status, education, income and working situation.

#### Baseline

Table 1 schematically shows the instruments used in the study. A structured diagnostic interview about the patient’s diagnosis is the first step in the baseline assessment. Axis I psychiatric disorders will be assessed by use of the electronic version of the MINI Plus (Mini Neuro-psychiatric Interview) [28]. The MINI Plus is the briefest full psychiatric interview available and takes, dependent on the number of disorders, between 15 and 45 minutes. A 10-item screening instrument will be used to assess whether a full structured diagnostic interview for Axis II psychiatric disorders is required. The Standardised Assessment of Personality – Abbreviated Scale - Self Report (SAPAS-SR) has been found one of the briefest, most sensitive and specific screening instruments for Axis II disorders [29]. We expect about 50% positive screens in this secondary care sample. A positive screen will be followed by the Structured Interview for DSM-IV (SIDP-IV) [30]. The SIDP-IV is a widely used semi-structured interview with good psychometric properties.

**Table 1:** **Measuring instruments**

Instrument	Measuring moments			
	T0(baseline)	T1(6 months)	T2(12 months)	T3(18 months)
**Filled in by researcher**				
Demographic questionnaire	X	X	X	X
MINI Plus	X			
SAPAS-SR	X			
SIDP-IV (when SAPAS-SR positive)	X			
CANSAS (Patient)	X	X	X	X
SNM	X	X	X	X
**Filled in by patient**				
OQ-45.2	X	X	X	X
MANSA	X	X	X	X
IMR (Patient)	X	X	X	X
EQ-5D	X	X	X	X
TiC-P	X	X	X	X
STAR (Patient)	X	X	X	X
**Filled in by professional**				
DDPRQ	X	X	X	X
HONOS	X	X	X	X
CANSAS (Professional)	X	X	X	X
IMR (professional)	X	X	X	X
STAR (professional)	X	X	X	X

All outcome measures (MANSA; HONOS; IMR; EQ-5D; OQ45; TiC-P; STAR; DDPRQ; CANSAS; SNM) will be assessed at baseline, and at 6 months, 12 months and 18 months. Referral to lower intensive services will be assessed at the 12-month and 18-month measurement.

### Primary outcome

#### Quality of life

Quality of life is measured on participant level with the MANSA [31]. The MANSA (Manchester Short Assessment of Quality of Life) is the single most used quality of life instrument for patients with severe mental illness. It is a 16-item patient-rated instrument with good psychometric properties.

### Secondary outcomes

#### Quality of life

The EQ-5D (EuroQol 5D) [32] is a patient-rated measurement of health-related quality of life, providing a generic measure of health for clinical and economic appraisal. It is applicable to a wide range of health conditions and treatments, and provides a single index value for health status that can be used in the clinical and economic evaluation of health care. It is a 5-item patient-rated instrument with good psychometric properties that allows the calculation of QALY’s and DALY’s.

### General mental health

The HONOS (Health of the Nation Outcome Scale) is a 12-item professional-rated instrument to assess general mental health in predominantly SMI-patients [33] with good psychometric properties and a mean duration of 10 minutes [34].

### Treatment

The OQ-45 (Outcome Questionnaire) a 45-item instrument which assesses treatment outcome, mostly in terms of symptom reduction [35] with very good psychometric properties and a mean duration of 10 minutes [36].

### Recovery

The Illness Management and Recovery (IMR) [37] scale was created to measure recovery outcomes produced by the IMR program. However, many other mental health care programs are now designed to impact recovery-oriented outcomes, and the IMR has been identified as a potentially valuable measure of recovery-oriented mental health outcomes in general. Psychometric properties were moderate and the scale has a mean duration of 10 minutes.

### Costs

The Tic-P (Trimbos/iMTA questionnaire for Costs associated with Psychiatric Illness) [38] measures direct costs of medical treatments such as the number of contacts with psychiatric services, the GP and multiple other care providers, psychometric properties are unknown and has a mean duration of 10 minutes.

### Referral to lower intensive services/primary care

Through administrative records it will be assessed to which extent patients are referred to lower intensive services, most likely primary care.

### Therapeutic relationship

The STAR (Scale To Assess the Therapeutic Relationship) [39] is a 12-item instrument that measures the quality of the therapeutic alliance between patients with severe mental illness and professionals. It is administered both by patients (STAR-P) and professionals (STAR-C), and has good psychometric properties.

The DDPRQ (Difficult Doctor Patient Relation Questionnaire) [40] is a 10-item instrument that assesses problems in the relationship between patient and professional and the perceived difficulty with very good psychometric properties.

### Care needs

The CANSAS (Camberwell Assessment of Need Short Appraisal Schedule) [22] is the single most used care needs assessment instrument among people with severe mental illness. Both the patient’s perception (through an interview by the researcher), and the professional’s perception (self-rated) are assessed through a 22-item checklist that measures met, unmet, and total needs for care.

### Social network

The Social Network Map (SNM) [41] is a researcher-assessed instrument using both a graphical (map) and textual (grid) instrument to assess the patient-perceived quantity and quality of his or her social network. The map is divided into sectors (household, other family, work/school, formal services, friends, neighbours, and clubs/organizations/church). The psychometric qualities of the instrument, as in all social network instruments, are acceptable.

### Statistical analyses

The primary outcome will be analysed using a linear mixed model (multilevel) to account for the nesting of clients within professionals and for the correlation over time of repeated measurements within subjects. The effect of ICPT versus CAU will be adjusted for important client and professional characteristics (e.g. quality of life) by including the latter as fixed effects in the model. Similar mixed models will be used to analyse the continuous secondary outcomes. All analyses will be performed on an intention-to-treat basis.

### Health economic evaluation

This study will investigate the potential efficiency of Interpersonal Community Psychiatric Treatment (ICPT) versus current long-term care (CAU) from a societal perspective. The economic evaluation will be based on the general principles of a cost-effectiveness analysis as described by Drummond et al. [42] and will be performed along-side the (cluster randomized) clinical trial. Outcome measures for the economic evaluation, considering the 18-months follow-up period, will be costs, quality of life and quality adjusted life years (QALYs). The cost analysis exists of three main parts.

First, on patient level, volumes of care will be measured prospectively using TiC-P (part I), administrative data. Cost items included are: number of outpatient contacts, home visits, number and length of hospitalizations, but also ER/casualty department-visits, ambulance transportation, and justice department contacts. Productivity losses for patients (sick leave) will be estimated using TiC-P part II. The friction cost-method will be applied following the Dutch guidelines for cost analyses [43]. Also travel time to an outpatient clinic and related costs patients make, will be considered. Second, the cost analysis consists of determining the cost prices for each volume of consumption in order to use these for multiplying the volumes registered for each participating patient. The Dutch guidelines for cost analyses will be used [44]. For units of care/resources where no guideline or standard prices are available real cost prices will be determined. Third, per arm (intervention and control) total costs will be determined using activity based costing. The effect analysis adheres to the design of a randomized controlled trial and measures at baseline, and follow-ups at 6, 12 and 18 months. To measure the quality of life of patients a validated so-called health-related quality of life (HRQoL) instrument will be used, the EuroQol-5D (EQ-5D) [45].

The incremental cost-effectiveness ratio’s (ICERs) ‘cost per unit change on the MANSA’ and ‘cost per QALY gained’ will be computed and uncertainty surrounding these ICERs will be determined using the bootstrap or Fieller method. Cost-effectiveness acceptability curves will be derived that are able to evaluate efficiency by using different thresholds (Willingness To Pay) for a unit change on the MANSA and a QALY gained. The impact of uncertainty surrounding deterministic parameters (for example prices) on the ICER will be explored using one-way sensitivity analyses on the range of extremes.

## Discussion

No RCT, nor cost-effectiveness study, has been conducted on Interpersonal Community Psychiatric Treatment so far – only one, small pilot study, promising better outcomes than in usual care. The empirical base for current care as usual is small, if not absent. This study will fill this void, and generate data that is needed to inform and hopefully improve daily mental health care. In summary, we assume that ICPT is more effective in improving patients’ quality of life and social networks, preventing or decreasing professionals’ perception of patients as ‘difficult’, discharging patients to a lower level of care and being less costly in reaching these clinical goals than CAU. The patient group we focus on, receives long term care and suffers from various non-psychotic psychiatric diagnoses. The strength of ICPT is its focus on this varied group of patients, for whom current care is unsatisfactory.

The rationale performing a cluster-randomized design is threefold. First: data from this trial will be clustered at multiple levels, due to three participating institutions, various departments within these institutions, and various professionals (=cluster level) within these departments. Second: contamination of treatment methods is likely when professionals treat patients in both the experimental and control condition. Contamination is less likely in the current design since participating professionals have limited contact on treatments or methods with one another outside official treatment progress meetings – in which there is little time to discuss treatment content. Therefore randomization at the level of professionals is preferable. Third: refusal to be randomized is likely with this patient group. Many patients find it difficult to switch to another professional since they may have a long-term working alliance with their present professional. Randomization therefore takes place on the professional level instead of on patient level.

It is expected that this study will yield results that may well be generalized across everyday mental health care. Since our target population consists of patients who are high care users, who are more willing to participate than patients who receive for example assertive outreach [9], we do not expect high selection bias. There are a limited number of inclusion and exclusion criteria in our trial. Unlike in many other trials, patients who are suicidal, aggressive, or self-harming are welcome to participate. Also, comorbidity as well as substance abuse are no exclusion criteria. To encourage participation by professionals, a tailored training-program was developed, based on their day-to-day work with the participants involved. A limitation of this study is the lack of blinding. Participating professionals will know that they conduct ICPT instead of CAU. Patients will also not be blinded.

## Abbreviations

CANSAS: Camberwell Assessment of Need Short Appraisal Schedule
CAU: care as usual
DALY: Disability-adjusted life years
DDPRQ: Difficult Doctor Patient Relation Questionnaire
EQ-5D: Europol 5D
HONOS: Health Nation Outcome Scale
ICER: incremental cost-effectiveness ratio
ICPT: Interpersonal Community Psychiatric Treatment
IMR: Illness Management and Recovery
MANSA: Manchester Short Assessment of Quality of Life
MHS: Mental Health Services
MINI Plus: (Mini Neuro-psychiatric Interview)
OQ45: Outcome Questionnaire45
QUALY: quality adjusted life years
SAPAS: Standardised Assessment of Personality –Abbreviated Scale, Self Report -SIDP-IV, Structured Interview for DSM-IV
SNM: Social Network Map
STAR: Scale to Assess the Therapeutic Relationship
Tic-P: Trimbos/iMTA questionnaire for Costs associated with Psychiatric Illness
